# "TOF2H": A precision toolbox for rapid, high density/high coverage hydrogen-deuterium exchange mass spectrometry via an LC-MALDI approach, covering the data pipeline from spectral acquisition to HDX rate analysis

**DOI:** 10.1186/1471-2105-9-387

**Published:** 2008-09-20

**Authors:** Pornpat Nikamanon, Elroy Pun, Wayne Chou, Marek D Koter, Paul D Gershon

**Affiliations:** 1 Department of Molecular Biology & Biochemistry, UC-Irvine, Irvine, CA 92697, USA; 2 Department of Parking & Transportation Services, UC-Irvine, Irvine, USA; 3 Department of Information & Computer Science, UC-Irvine, Irvine, USA

## Abstract

**Background:**

Protein-amide proton hydrogen-deuterium exchange (HDX) is used to investigate protein conformation, conformational changes and surface binding sites for other molecules. To our knowledge, software tools to automate data processing and analysis from sample fractionating (LC-MALDI) mass-spectrometry-based HDX workflows are not publicly available.

**Results:**

An integrated data pipeline (Solvent Explorer/TOF2H) has been developed for the processing of LC-MALDI-derived HDX data. Based on an experiment-wide template, and taking an *ab initio *approach to chromatographic and spectral peak finding, initial data processing is based on accurate mass-matching to fully deisotoped peaklists accommodating, in MS/MS-confirmed peptide library searches, ambiguous mass-hits to non-target proteins. Isotope-shift re-interrogation of library search results allows quick assessment of the extent of deuteration from peaklist data alone. During raw spectrum editing, each spectral segment is validated in real time, consistent with the manageable spectral numbers resulting from LC-MALDI experiments. A semi-automated spectral-segment editor includes a semi-automated or automated assessment of the quality of all spectral segments as they are pooled across an XIC peak for summing, centroid mass determination, building of rates plots on-the-fly, and automated back exchange correction. The resulting deuterium uptake rates plots from various experiments can be averaged, subtracted, re-scaled, error-barred, and/or scatter-plotted from individual spectral segment centroids, compared to solvent exposure and hydrogen bonding predictions and receive a color suggestion for 3D visualization. This software lends itself to a "divorced" HDX approach in which MS/MS-confirmed peptide libraries are built via nano or standard ESI without source modification, and HDX is performed via LC-MALDI using a standard MALDI-TOF. The complete TOF2H package includes additional (eg LC analysis) modules.

**Conclusion:**

"TOF2H" provides a comprehensive HDX data analysis package that has accelerated the processing of LC-MALDI-based HDX data in the authors' lab from weeks to hours. It runs in a standard MS Windows (XP or Vista) environment, and can be downloaded  or obtained from the authors at no cost.

## Background

Polypeptide backbone amide proton-deuterium exchange (HDX) analysis provides a powerful approach for understanding protein backbone solvent accessibility and conformational change. Despite the availability of NMR-based HDX methods, the MS-based approach is sensitive and amenable to larger molecules (albeit exchange rates are not always assignable to individual amino acid residues). In MS-based HDX, exchange rates are not analyzed in real time. Instead, individual timepoints are quenched from a deuterium exchange reaction with cold acid then each is incubated with an acid protease (typically pepsin) and the resulting peptides are rapidly resolved and analyzed via LC-MS then identified by correlation with prior LC-MS/MS experimental data. To minimize back exchange (BE) of acquired deuterons with solvent protons during wet sample processing, low temperature and low pH conditions are maintained after quenching, and wet analysis should be rapid.

The generation of potentially very complex peptide mixtures by digestion with a low-specificity protease necessitates LC analysis with dynamic range and resolution, which should nonetheless be speedy in order to minimize BE. Minimizing potential ambiguities in peptide assignment within mass spectra from complex chromatograms requires accurate mass measurement, MS/MS on the fly, use of the reversed phase retention time parameter and/or fitting of experimental to candidate theoretical isotope distributions. Our current path around these challenges has been a combination of the above, namely mass-accurate MALDI-TOF analysis in combination with the rapid reversed phase LC nano-fractionation of HDX experimental samples. This is preceded by a series of preliminary LC-MS/MS analyses in order to build, then saturate, a non-redundant peptide library (done via an LC-MALDI-TOF/TOF approach). Finally, in place of simple LC retention time analysis we correlate "Z" number (critical organic modifier concentration) [1] between HDX and MS/MS library experiments, for each peptide irrespective of LC instrument or solvent mixture.

To our knowledge, the use of MALDI for HDX work was first reported by the Komives lab, with the introduction of features such as immobilized pepsin for digestion and pre-chilled MALDI plates [2]. Additional reports built upon this approach [2-5]. However, these experiments were done with unfractionated peptide mixtures and, to our knowledge, there have been no reports of HDX by LC-MALDI. Strengths of MALDI-TOF/TOF as a platform for HDX work would be expected to include accurate mass determination (with internal calibration), spectral resolution for accurate spectral peak area analysis/centroiding and the absence of a heating step during sample introduction into the gaseous phase. Being an offline technique, mutual exclusivity is maintained between sample fractionation and acquisition speeds, and between chromatographic and spectral resolution. This contrasts with some online instruments, in which the slower scan speeds required for good spectral resolution may place a practical limit on both LC gradient times and the high chromatographic peak resolution required to prevent peptide-to-peptide spectral interference and undesirable overlap of the deuterium-shift trajectories of peptides in crowded spectra – with crowding being a particular hazard at the high dynamic ranges achievable with such instruments. Finally, TOF-based work has the well-known advantage of yielding essentially singly charge ions as the sole species in peptide work, simplifying data interpretation. Thus HDX workflows play to many of the well-known strengths of MALDI-TOF-based technology. For our "HDX by LC-MALDI" analysis, we employed the ABI 4700 MALDI-TOF/TOF mass spectrometer for data acquisition. In our hands, samples can be in the instrument within 12 min of deuterium uptake quenching (2 min for protein digestion, 1 min for column injection followed by a 3 min LC gradient within a 5.5 min spotting window, 20 sec for sample drying, and 15 sec to dock the plate within the instrument). Within the gaseous phase within the instrument, in which BE is finite but in our hands an order of magnitude slower than at atmospheric pressure, MS data from a set of 192 spots can be fully acquired within 30 minutes of plate docking.

For HDX experiments, specialized software tools are required to reduce comprehensive datasets to manageable proportions. Minimally, target peptides should be identified within mass spectra that may be numerous and crowded, from which the corresponding target ^13^C/^2^H isotope clusters must be excised, then the resulting spectral segments averaged across an XIC peak and analyzed for deuteric mass shift. Tools have been reported which address part or all of such workflows. These include: "AUTOHD" [3], which fits measured isotopic distributions to modeled distributions calculated via fast Fourier transform, and includes a module for isotopic deconvolution of mass spectra (or of non-deisotoped instrument software-derived peaklists), correlating clusters with peptides based on accurate mass and isotopic distribution. However, this software is designed for use with infused samples only (non-LC fractionated). More recently, "DEX" from the Komives lab [4] provides a tool which appears to have essentially the same function as AUTOHD. Weis & Engen's "HX-Express" [5] also covers the latter part of the standard HDX workflow, namely calculating centroid masses for isotope clusters corresponding to chosen peptides, excised from raw spectra in ASCII text format, followed by the generation of deuterium uptake rates plots as Microsoft Excel charts. Calculations are based on weighted mean mass of the isotope cluster rather than fitting to a theoretical distribution.

Scripps-Florida's "The Deuterator" [6] (recently updated to HD Desktop – [7]) was a first attempt to automate the processing of HDX data from scanning mass spectrometers, including steps prior to isotope cluster analysis. The workflow for this software includes the initial curation of MS/MS-confirmed peptide data, the automated editing of LC-MS spectral stacks from HDX experiments as delimited by the positions of HDX isotope clusters predicted from the initial curation, averaging of the stacked spectral segments, and isotope centroiding/fitting to the segment average. In the first step, peptides whose independently acquired MS/MS spectra match the protein of interest in independent database searches (eg. Sequest, Mascot [8], X!Tandem) are manually curated (on the basis of either strength of score or protein region of interest) in a listing that includes peptide sequence and a chromatographic retention time range. In the second step, a retention time-range/mass-range "box" enclosing the mass spectral cluster assigned to a peptide sequence in MS/MS experiments guides the automated editing of spectral stacks arising from LC-MS analysis of HDX timepoints. After averaging of the stacked segments and removal of spectral peaks attributable to interfering clusters, the segment average is subjected to deuterium uptake calculations by either least-squares fitting of theoretical isotope distributions to the observed cluster [9], or centroid (weighted mean) calculation [10]. "The Deuterator" has a spectral viewer that displays peptide information (as text) along with XICs and spectral data. Deuterium buildup curves can be plotted using software such as MS Excel.

At the time thta HDX work was initiated in the authors' lab (prior to availability of "The Deuterator", above), no automated tools were available for the processing of HDX data including steps prior to isotope cluster analysis. Moreover, as a result of our novel LC-MALDI approach to HDX sample analysis, which involves off-line peptide fractionation followed by analysis in a MALDI-TOF instrument, data acquisition and processing requirements are significantly different from those that are commonly used, in terms of templating of the experiment and the qualities/numbers of spectra produced. We therefore developed a series of macros into a robust toolbox ("TOF2H/Solvent Explorer") for HDX analysis via LC-MALDI MS, and these are described herein.

## Results and discussion

### Raw data format in the AB-SCIEX 4700 MALDI-TOF/TOF mass spectrometer

The AB-SCIEX 4700 MALDI-TOF/TOF mass spectrometer acquires mass spectra (plots of m/z (x) vs. intensity (y)) for samples spotted/dried onto stainless steel plates. The standard "Opti-TOF" plate used by the authors for HDX experiments accommodates up to 192 samples deposited within pre-etched spots denoted according to rows (A – H) and columns (1 – 24) with each having a unique alphanumeric "Spot_label". Via the manufacturer's "4000 Series Explorer" software, the AB-SCIEX 4700 is controlled, and mass spectra are acquired from individual spots then stored in an Oracle database ("TSQUARED"). TSQUARED is routinely accessed via "4000 Series Explorer", leading to the display, within a graphical user interface (GUI), of a central table ("spotset"), each row of which corresponds to a spot on the sample plate. Upon docking a plate that has been spotted for the first time (or for the first time after cleaning), a new spotset ("spotset_ID" in TSQUARED) is initiated.

Each spectral acquisition/storage event in the AB-SCIEX 4700 generates and stores in TSQUARED: (**1**) A "raw spectrum", ie. a lengthy encoded list of m/z-intensity pairs to be joined in displayed spectral plots, (**2**) An "output peaklist", ie. a tabulated listing of the properties of all peaks detected in the raw spectrum ("Centroid Mass", "Height", etc.) as interpreted by instrument-based spectrum processing methods with user-adjustable parameters. In the "4000 Series Explorer" software, each primary peaklist (ie. a comprehensive list of spectral peaks exceeding a specific, preset S:N threshold) is treated to reduce clusters of peaks attributable to naturally-occurring isotopes of molecules with a specified molecular formula (eg. C6H5NO – the approximate molecular formula of a generic peptide) at natural abundance, to just one peak, namely the monoisotope. As far as we could ascertain, only the resulting listing, named the "output peaklist" is available to the user, eliminating the opportunity for theoretical isotope fitting to instrument-derived peaklist data during HDX experiments. Groups of spectra within a spotset are denoted as linked via a shared five-digit Job_run_ID (JRID), which is unique within TSQUARED (and ascends incrementally, chronologically and practically indefinitely). A new JRID is assigned whenever a new automated (batch) data acquisition session is initiated. Thus, in HDX experiments for example, all LC-MALDI spots for a particular HDX timepoint are associated with one another via a shared, unique JRID.

### Preliminary MS/MS experiments and per-experiment MS/MS-confirmed peaklist (MPL) files

**Figure 1:**
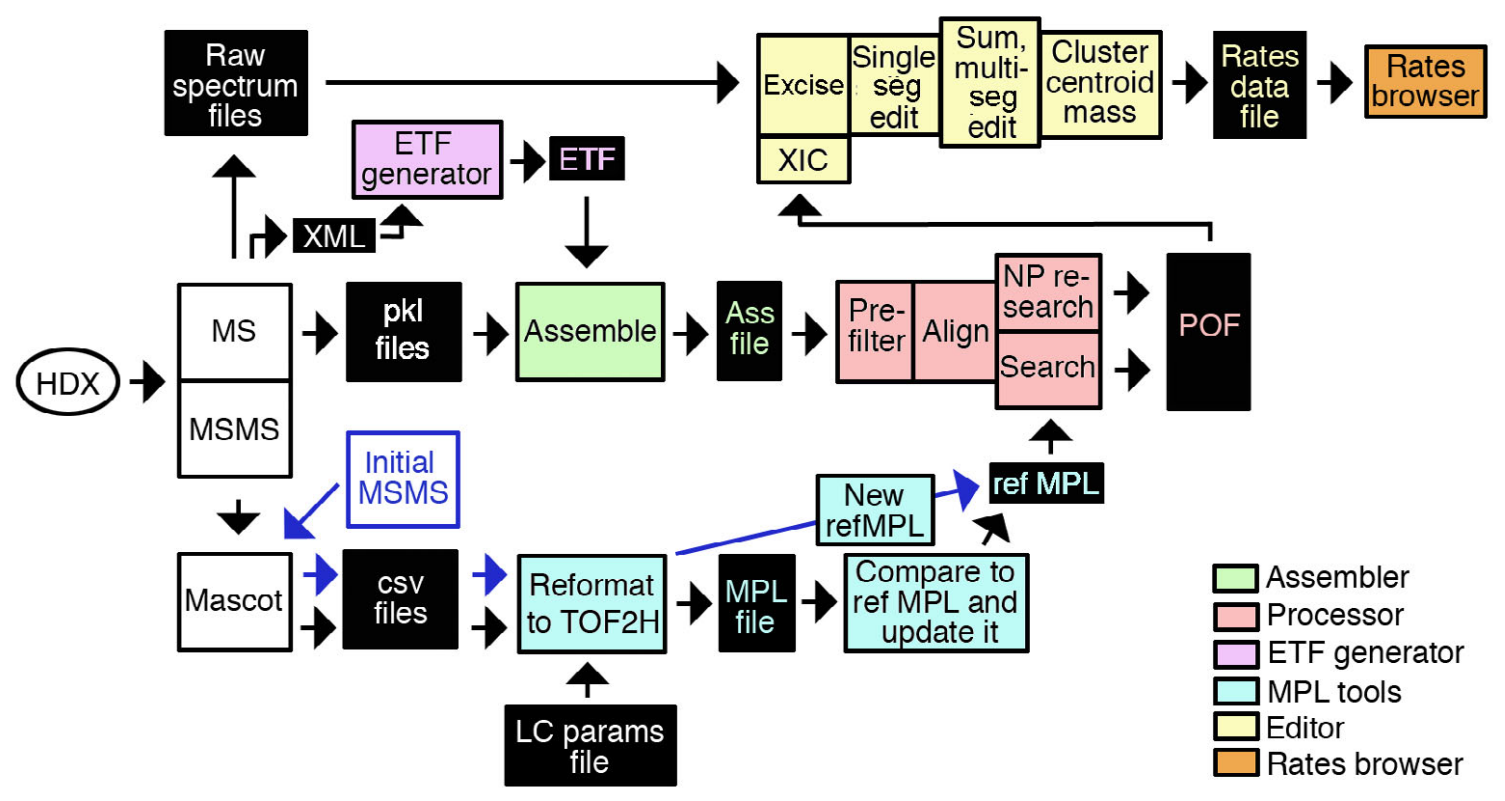
**Data flow within TOF2H.** See text for details.

The flow of experimental data through TOF2H is shown in Fig. 1. The directory structure employed by TOF2H at the time of writing (as setup by the "TOF2H-New experiment" macro) is shown in Additional file 1, in which the experiment series level contains project-specific information relevant to all experiments having a particular target protein/protease combination. File types and functions are indicated in Additional file 1.

At the outset of a new experiment series or project, preliminary nanoLC-MALDI MS/MS experiments are performed (see "METHODS") in order to build a non-redundant library of MS/MS-confirmed peptides for the target protein/protease of choice. Via TOF2H's "MPL curation tools" macro, the database search output from each MS/MS experiment, exported in .csv format, is reformatted to TOF2H's "MPL" format (mainly to allow the addition of HD Exchange information and fractionation/LC parameter information for each peptide, (Additional file 1: Footnote 1), with one MPL workbook per experiment containing one MPL sheet per protein hit in the .csv. To the MPL workbook are attached the original .csv and a manually-filled spreadsheet detailing LC gradient and fractionation parameters for the overall MS/MS experiment. LC fraction numbers are converted between templated and actual spotting patterns where necessary to defeat the more limited range of spot pattern templates available within the mass spectrometer software. A Mascot "expect" = 0.05 cutoff is typically employed for inclusion in the MPL. The resulting MPL workbooks are stored at the "Experiment series" level within the directory hierarchy (Additional file 1) since they are relevant to all subsequent experiments with specific protease and target polypeptide.

### "Reference MS/MS-confirmed reference peaklist" (refMPL) – a "dynamic", non-redundant compendium of MS/MS data for a specific protease/target protein

While MPL files are generated on a per-MS/MS-experiment basis, the refMPL (reference MS/MS-confirmed peaklist) is a non-redundant compendium of all MS/MS confirmed peptides for a specific protease/target protein combination, derived from multiple MPLs. The refMPL is structurally distinct from precursor MPLs in that it (**a**) contains only MPL sheets (no LC parameters data, etc), and (**b**) is continuously updatable ("on the fly") with MPL sheets from additional rounds of MS/MS analysis of the same specific protease/target protein (such as, the undeuterated ("F1H") controls of additional HDX experiments, as they become available). For a round of updating, MPL files/sheets are selected individually for comparison with all extant data in a chosen refMPL using "TOF2H-MPL curation tools"'. A difference or "Comparison" sheet is generated, which can be transferred to the refMPL as an "Update" sheet. All such operations are automatically time-stamped to preserve the chronology of progressive updating. RefMPL contents can be displayed in sequence-contig format using a contig. viewing tool, showing also the % sequence coverage and the positions of any inter-contig breaks.

### Implementation of an HDX wet experiment, Data Extraction from TSQUARED, Experiment Template File

Next, HDX timepoint experiments can be implemented as described in "Methods". For each HDX experiment, a unique "experiment template file" (".etf" or "ETF") contains an "experiment template" (ET) spreadsheet indicating the overall layout of the LC-MALDI HDX experiment (Table 1, Additional file 1). The ET sheet indicates, also, all spots that failed internal calibration during data acquisition ("Def Cal" spots). Information in the ET sheet co-ordinates data assembly, largely via the construction/deconstruction of file paths and extracted datafile names. Additional spreadsheets within the ETF include layout map(s) for all MALDI target plates used, and tables for relevant LC gradients.

**Table 1:** Header and initial few lines of an ET (Experiment Template worksheet, vertical-format).

**Spot_Set_Name**	**Timepoint**	**Gradient**	**Fraction**	**spot_label**	**Def cal?**	**JR**	**Job_run_ID**
2007-12-02_VP55_5min_Capillary	900 sec	1	1	A1	**D**		28998

			2	A3	**D**		

			3	A5			

			4	A7			

			5	A9			

			6	A11			

			7	A13			

			8	A15			

			9	A17			

			10	A19			

			11	A21			

			12	A23			

			13	A24			

			14	A22			

			15	A20			

			16	A18			

The complete table contained 2688 data rows (total LC fractions). The ET cross-references experiment-specific parameters (numbers of timepoints, number of fractions in each timepoint, actual deuterium exchange times, LC gradient type, spot label for each individual fraction number generated via one of four embedded spot pattern generators), with acquisition-specific parameters (spotset names, JRID values and spot label for every spectrum and every output peaklist associated with the experiment). A horizontal format (not shown) is also available for smaller experiments (for fewer than 63 total fractions as limited by the 256 column limit of MS Excel 2003). Column "JR" is currently unused.

After recovery of peaklist and raw spectrum data from TSQUARED (above), the ETF is built *de novo *by"TOF2H-ETF Generator". Prior to building, user-inputs comprise: Experiment series name/experiment name, relevant spotset dumps, ETF format (horizontal/vertical), preset MALDI spotting pattern, whether spot labels are alphanumeric or numeric, and confirmation of the macro-deduced timepoint/BE timepoint corresponding to each job run ID. "ETF Generator" then assesses the contents of the T2DE data dump, detecting: (**a**) multiple acquisitions of a spot; (**b**) aniticpated LC-MALDI fractions missing in data dumps; or (**c**) multiple JRIDs per timepoint. Thereafter, cross-correlations are deduced automatically between spotset, spot, HDX timepoint and LC fraction (Table 1)). All fractions whose acquisition failed internal calibration are then marked on the ET sheet using, as a guide, an XML-formatted summary of all acquisition parameters for the experiment exported from TSQUARED. Alternative ETF generation tools return various levels of manual control to the above scheme (as may be useful for smaller experiments and/or non-conventional experimental formats).

### TOF2H-assembler: Import and assembly of all MS peaklist data into a single worksheet

**Figure 2:**
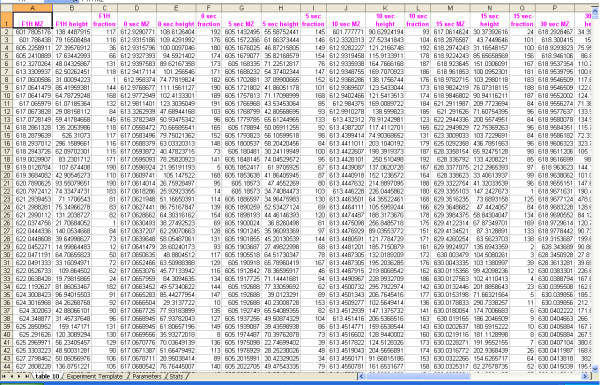
Screenshot showing upper left segment of assembled mass/peak height/fraction data table (the complete table contained 168,000 data triplets).

ETF generation is followed by peaklist assembly. Here, "TOF2H-assembler" compiles all monoisotopic m/z-intensity values from the set of dumped output peaklist (.pkl) ASCII text files for the experiment into a single worksheet, using the .etf as a guide (typically ~30,000 to ~170,000 data triplets from thousands of .pkl files). Each m/z-intensity pair is tagged, on the fly, with LC fraction number, and the resulting data triplets are listed in a single column triplet per timepoint/BE timepoint, sorted by ascending mass. Column triplets are horizontally juxtaposed by ascending deuterium uptake time (bracketed by F1H and F2H and followed by ascending BE time). The resulting spreadsheet is supplemented with a marked-up copy of the ET sheet, spreadsheets for recording program run parameters and statistical output (below). The resulting assembler output (".ass") file comprises the comprehensive MS peak summary for the experiment (Fig. 2).

### TOF2H-processor: Pre-filtering steps (sheets 10, 15, 20, 25, 30, 35)

The assembled peaklist spreadsheet of the .ass file is processed, sequentially, through a series of steps by "TOF2H processor", the output of each being preserved as a precursor for the subsequent step (Table 2). The .ass file spreadsheet is first "pre-filtered", followed by the alignment of equivalent masses between timepoints, followed by a library search for peptides relevant to the target protein and for deuteration-dependent isotopes of library hits. Results are written to an output spreadsheet. In our hands, the above steps are able to reveal exchange-active peptides of target proteins from instrument-derived output peaklist data alone (without spectral editing or isotope fitting). Pre-filtration/mass alignment and library search steps may be coupled or run independently.

**Table 2:** Glossary of spreadsheets in TOF2H-Processor workbook (sheets numbered in increments of 5 to accommodate future development)

**Table**	**Function**	**Operation**	**Preliminary columns**	**Columns per timepoint**	**Columns**	**Notes**
Sheet 10	Assembled peaklist data (same as assembler output file)	Copy of assembled Peaklist data		m/z/peak height/identifier		Each set of columns (timepoint) is ranked by ascending m/z

Sheet 15	Highlighting of Def Cal spots masses	Def Cal data highlighted		m/z/peak height/identifier		

Sheet 20	Deletion of Def_cal spots masses, highlighting of all isotopomers	After Def Cal mass deletion, isotopomer rungs/monoisotope colored correspondingly, rungs indexed to monoisotope, and match mass error values for each isotope indicated in an additional column		m/z/peak height/identifier/monoisotopeIndex/isotope mass error		

Sheet 25	Removal of all isotopomers, highlighteing of calibrant masses	Sheet 20 without isotopomers; color info removed, then calibrant masses colored		m/z/peak height/identifier		

Sheet 30	Filtering of calibrant masses	Copy of Sheet 25 with calibrant masses removed		m/z/identifier		

Sheet 35	Selection of XIC peak only for each peptide	Copy of XIC peak-fraction info only for each peptide in Sheet 30		m/z/identifier		

Sheet 40	Cluster finding				Sheet 45 cluster start row/Sheet 45 cluster end row	

Sheet 45	Collation of sheet 35 into a pair of columns with column of origin in third column. Row assignment for all peptides in fourth column padded according to Sheet 40	Collate Sheet 35 into column triplet, find mass group boundaries, assign new row identifiers			m/z/identifier/substitute assigned row/assigned column	

Sheet 50	Sheet 35 after padding according to Sheet 45 row assignments			m/z/identifier		

Srch1	Sheet 45 hits to refMPL, plus candidate isotope progeny, copied from Sheet 45		Query mass/spot ID/peptide info/primary mass tol/progeny mass tol	m/z/identifier		

Srch1P	Search parameters					

Filt. Params	Pre-filtration parameters					

Stats	Enumeration of data					

**Figure 3:**
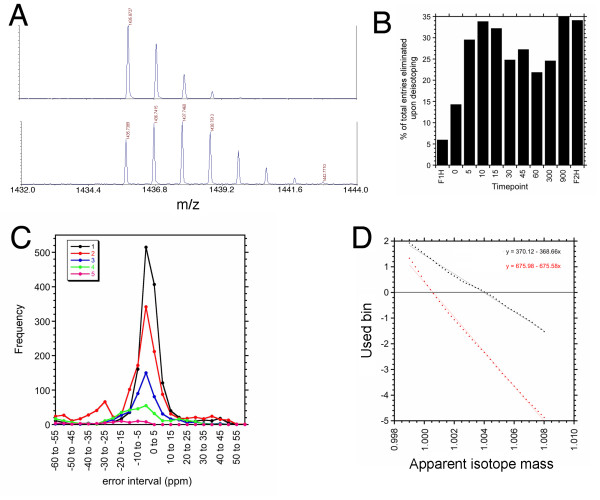
**Deisotopingof peptide-clusters.** (**a**) m/z 1425 peptide in MALDI-TOF spectra from two samples: Upper – F1H (fully 1H; instrument-deisotoped). Lower – after deuteration (incompletely instrument-deisotoped) (**b**) Count of discarded isotope masses in the timepoints from a typical deuterium exchange experiment. F1H and F2H are fully protic and fully deuteric, respectively, other times are in sec. F1H isotope discovery rate was typically 1% that of F2H (data now shown). The discovery of some isotopes in F1H could be attributed to the occasional occurrence of perfectly coincident fully protic peptides, upsetting the deisotoping algorithm in the AB 4700 mass spectrometer software, or closely apposed spectra to within fractional mass. (**c**) Histogram (line display format) showing distribution of isotope mass errors among the 3670 total isotopes detected with isotope mass error tolerance set to +/- 60 ppm, deisotoping gradient factors = 0 (no incremental widening of tolerance per isotope increment). Number of "rungs" (isotope increments) </= 5. From this histogram, +/- 12 ppm was chosen as the default deisotoping mass tolerance, with positive and negative gradient factors both set to 1 (one doubling of mass tol per additional isotope) and five rungs max. In a random sample dataset, TOF2H picked 183 out of the 189 isotopic peaks that were identified by eye, ie a correct hit rate of 96.8% (3.2% false negative). (**d**) Determination of an isotope mass difference that nulls the distribution in (c). In each of the two experiments, a series of test isotope masses (panel c, all rungs combined) was tested followed by calculation of the weighted mean bin-point (y) of the resulting histograms and linear regression of each series. The optimal inter-isotope mass for each experiment is the value of x where y = 0.

The goal of the pre-filtering steps was the achievement of just a single data triplet for each peptide detected with accurate-mass in each timepoint. In the initial filtering step, spots failing internal mass calibration (the so-called "DefCal" spots) are highlighted then discarded, using the ET as an index. This step was imposed to prevent the errant misinterpretation of equivalent peaks as independent ones during the inter-timepoint mass alignment steps (below), as was found to occur in early versions of TOF2H. The second pre-filtering step is a de-isotoping one, imposed in order to pick up deuteration-time-dependent isotope shifts in the final search output, which would otherwise be masked. Although output peaklists from 4000 Series Explorer software had undergone deisotoping for natural abundance elemental isotopes known to occur within peptides, the superposition of an artificially-induced H-D isotope distribution led to a breakdown of the instrument's deisotoping function, and the consequent retention of many isotopomers in the instrument's output peaklist files (Fig. 3a). This effect was not unique to the AB 4700 MALDI-TOF/TOF's instrument software: In our hands, MatrixScience's "Mascot Distiller", for example, was also unable to fully deisotope distributions from artificially deuterated samples (not shown). The aim of full deisotoping was not to find monoisotopic peaks, but rather, to reduce the number of list entries per isotope cluster to just one (the lowest detectable mass peak for the distribution).

The deisotoping algorithm is not a fitting function. Instead, it simply detects the inter-isotope mass +/- deisotoping mass tolerance, up to a user-settable maximum number of isotope increments, with "gradient factors" to permit the progressive broadening of deisotoping mass tolerance with number of isotope increments (all values adjustable). The discarded isotope mass count increased with experimental deuterium uptake time (Fig. 3b), validating the algorithm in practice. Upon checking a subset of ~200 of the discarded isotope masses manually against the corresponding raw spectra, false positives (discarded masses that were actually superimposed, overlapping or neighboring peaks with comparable fractional mass) and false negatives (genuine isotope masses that were ignored) were very infrequent (Fig. 3 legend). Regarding the former, closely-apposed cross-talking mass clusters may be of limited value for HDX studies anyhow, in the absence of theoretical fitting [3,4]. The very occasional occurrence of false negatives could be regarded as an unavoidable statistical outlier phenomenon. Since isotopes include induced deuteration at various levels, superimposed over the mixture of ^13^C, ^2^H, ^15^N, ^17^O, ^18^O, ^33^S, ^34^S and ^36^S isotopes found in peptides at natural abundance (all with different nuclear binding energies), TOF2H-Processor can generate dataset-specific frequency distributions of experimental deisotoping mass errors for a given isotope mass difference (Fig. 3c), then empirically determine the isotope mass difference that nulls the distribution (Fig. 3d). The resulting value can then be used for deisotoping as an alternative to the standard H-D mass difference (eg. 1.006276744 amu, [4,11] or 1.0057 Da (anonymous referee)).

In the penultimate pre-filtering step, TOF2H-Processor highlights then deletes internal calibrant masses. In the final pre-filtering step, XIC clusters (equivalent m/z values from different spots within the same LC gradient) are located and, within each XIC group, only the data triplet with greatest intensity is retained. Mass tolerances used for this and subsequent mass-equivalence determination steps are described in Additional file 1.

### TOF2H-processor: Inter-timepoint mass matching and alignment (sheets 40, 45, 50)

Mass/height/fraction triplets surviving the pre-filtering steps are subjected to mass correlation between timepoints. This is done by copying values for all timepoints to a single column triplet, which is then searched for mass group boundaries. Each mass group is then dispersed back across the timepoint domain after calculating spreadsheet row numbers to bring equivalent masses into alignment.

### TOF2H-processor: HDX mass matching to MS/MS-confirmed peptide libraries

**Figure 4:**
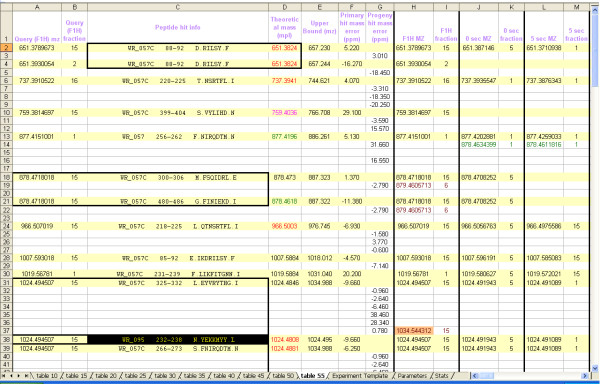
**Screenshot showing upper left segment of TOF2H-Processor output.** Columns B – E, comprising query peptide info (m/z, fraction, accession/endpoints/sequence), theoretical mass and "upper bound" contain data imported from the refMPL. Column D coloration indicates refMPL sheet of origin. Any adduct hits (not shown) are recorded with blue text coloration of the peptide info cell with adduct info stated. Columns F – G show mass errors. Remaining columns show mass-matching HDX timepoints data. Yellow bars denote primary hits, green masses in deuterium-uptake timepoints show "isotope progeny" of the primary hit masses (progeny were more plentiful in the longer timepoints further to the right, not shown). For isotope progeny searching, the search algorithm can progressively relax mass tolerances with increasing theoretical isotope increments. Brown masses are "false progeny". True progeny listed beneath a false progeny in timepoint samples, are typically isotope progeny (deuteration) of the false progeny. Salmon background denotes the progeny mass that was initially hit from among those on the same row. Inverted coloration (yellow text against black background) indicates a decoy hit. Black borders surround ambiguous hits (either more than one query hitting same theoretical mass (eg. upper box), or a single query hitting more than one theoretical mass (eg. center box). Good forward exchange/relatively low BE is indicated by increased spacing between yellow bars (more numerous progeny, out of view). Inferior results (not shown) would be immediately apparent as fewer progeny (little or no spacing between yellow bars).

Next (or independently), TOF2H queries the chosen refMPL files for F1H theoretical MH^+ ^matches to each HDX-F1H monoisotopic MH^+ ^value in turn. In the event of HDX experiments having mixed, multiple-protein targets, multiple refMPL files can be simultaneously searched. To search for cross-matching peptide masses from a non-target protein within a protein mixture, a refMPL may alternatively be selected as a "decoy" and searched only upon finding a hit within the target protein's refMPL. Within any chosen refMPL, sheets may be searched optionally (eg. update sheets only). Upon finding a match, the entire matching row from the row-aligned-mass table (above) is reconstructed in the nascent search result table (Fig. 4, yellow background) along with peptide information from the refMPL and search mass error. If the hit is a decoy, this is denoted with specific background coloration in peptide info cell. Ambiguous hits (multiple query peptides matching a reference mass or vice versa) are boxed (Fig. 4). Theoretical masses corresponding to peptide adducts (from within a user-defined adducts group) may be included in a search.

### "Isotope-progeny" and "False progeny" check (sheets 45, 50)

At later timepoints in HDX experiments, the shift in a peptide's isotope envelope may be sufficient to drive the abundance of the monoisotopic peak below the S:N threshold employed for peak detection (above), so that the first isotope listed for an isotope cluster is not the monoisotope. To reveal such clusters, TOF2H-processor performs, for each primary hit (above), an on-the-fly "candidate isotope-progeny check". Specifically, all entries in the pre-filtered mass list possessing mass values between the hit mass and "upper bound" (the calculated upper limit for possible deuteric shft) are scanned for matches to isotopic increments of the hit mass. Upon finding a candidate isotope progeny hit, the corresponding row of the aligned-mass table is reconstructed in the nascent search result table, directly beneath the primary hit (Fig. 4). The search results table thus provides an immediate visual indication of the extent of deuteric shift (or absence of BE) for each experimental peptide detected, along with the deuterium uptake timeframe of the shift, prior to any spectrum editing. The absence of progeny does not indicate an absence of deuterium uptake, simply insufficient uptake to send the monoisotopic peak into the noise.

If the progeny hit has a corresponding value in the F1H column, then this mass is marked as a "false progeny" (the F1H timepoint has never been exposed to deuterium). False progeny almost invariably arise from a closely juxtaposed peptide cluster whose fractional mass places it within the mass tolerance window. Such a situation may arise because the (intra-spectrum) deisotoping mass tolerance is set narrow (~12 ppm) over just 5 isotope increments, yet progeny may be sought at "inter-plate" mass tolerance (~40 ppm, Additional file 1) over a "theoretical isotope shift space" that extends to upper bound (which, for a 1000 Da peptide, may be ~10 increments).

At the conclusion of each processing stage (data assembly, pre-filtering and search), parameter sheets are time-stamped and updated with user-adjustable settings, pathnames for input files and program versions, and all table contents enumerated. Multiple searches can be conducted and stored per Processor output file.

### TOF2H-Editor

**Figure 5:**
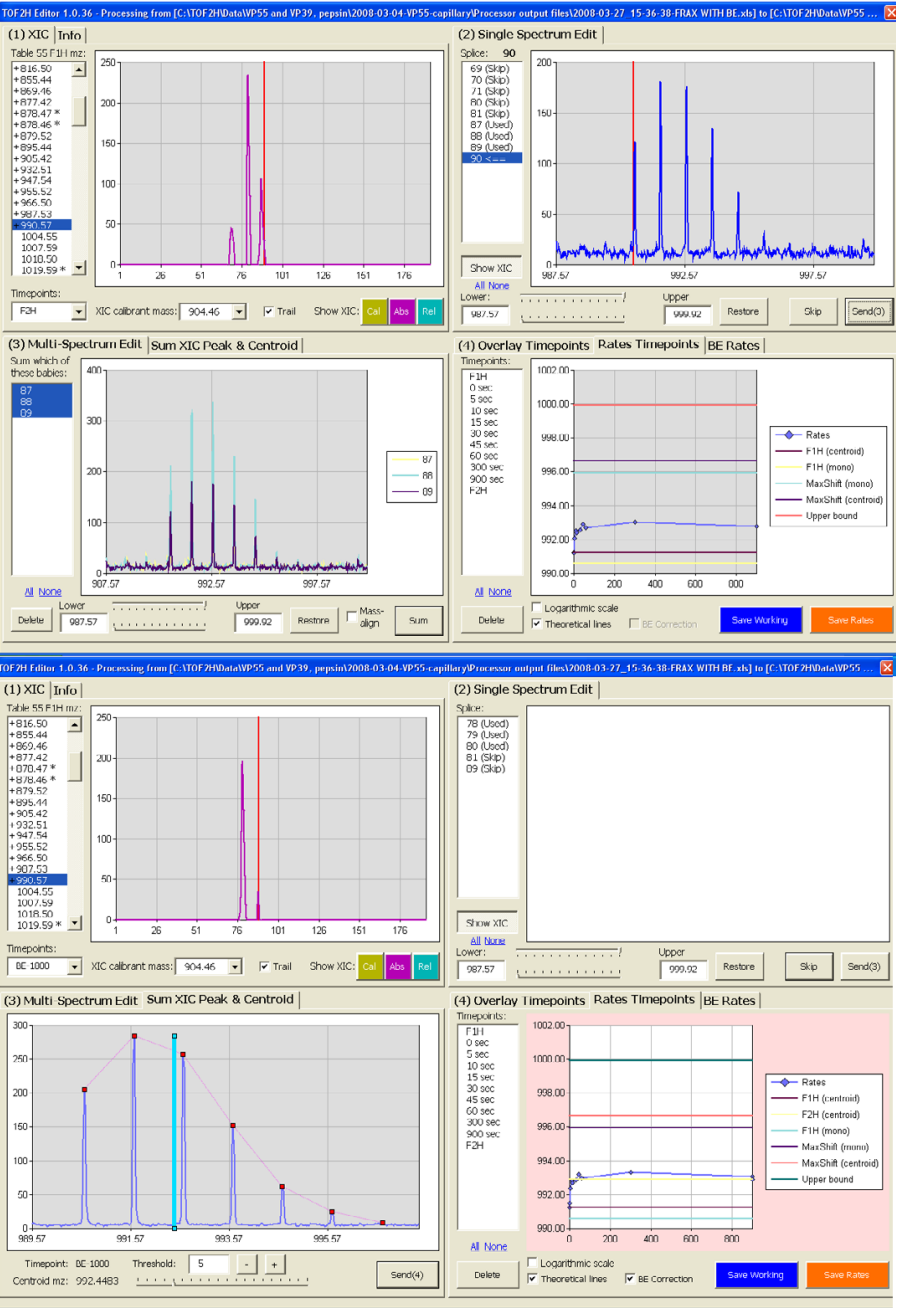
**Screen captures of TOF2H-Editor GUI.** In each of the two screen captures shown, upper left, upper right, lower left, lower right quadrants (Q1, Q2, Q3, Q4, respectively) are as described in the text. In the peptide listing (upper left), already-processed entries are prefaced with a "+"). For the XIC displayed (Q1), active XIC fractions populate the listing in Q2, and the spectral segment for the first is plotted (from 3 Da below F1H monoisotopic mass to either "upper bound" or the highest mass isotope detected). In Q2, spectral segments can be sequentially rejected or sent to Q3 (after re-endpointing if desired, though upon sending to Q3, overlaid segment lower endpoints default to 0.5 Da below the monoisotopic mass). In win(3), segments can be mass-aligned (via a small offset to slight mass errors), then averaged, leading to the view in the lower panel in which spectral peaks are detected then the weighted mean (centroid) mass calculated. Sending the centroid mass to Q4 adds a point to the growing rate plot (shown with overlaid theoretical lines). In addition, the averaged raw spectral segment is sent to another tab of Q4 (not shown). Q4-upper, lower: Before and after BE correction, respectively.

TOF2H-Editor is an ergonomic tool for developing deuterium uptake rate plots from raw spectra using the TOF2H-Processor search results as a guide with real-time spectral segment validation. A four-quadrant multi-tabbed GUI was developed whose quadrants 1, 2, 3 and 4 contain charts for: (1) XIC; (2) Single spectrum editing, (3) multi-spectrum edit/sum XIC peak/peak-detect/cluster-centroid, (4) BE-correctable deuterium-uptake rate plot/BE rate plot. Rates plots are built progressively across the deuterium uptake timepoints for each peptide) (Fig. 5). Editor workflow was streamlined with a target throughput of < 3 min processing time per peptide in a 14-timepoint experiment (11 experimental plus three BE timepoints) with real-time validation of all data.

In TOF2H-Editor, windows are tabbed to allow the viewing of main and alternative charts in each. In the standard workflow, masses corresponding to search hits (above) are selected from a listing in quadrant 1 (XIC, upper left). For the selected peptide and timepoint, the XIC is then displayed in quadrant 1, above-noise XIC fractions are highlighted in a listing in quadrant 2 (upper right), and a spectral segment for the first of these is read from the corresponding raw spectrum ASCII and displayed in quadrant 2. "Upper bound' (above) is used as the initial upper endpoint mass for the segment. TOF2H-Editor recommends acceptance/rejection of the segment and, minimally, the operator visually approves or rejects the segment via buttons under the quadrant 2 spectral plot, or allows TOF2H-EDITOR to accept/reject without intervention. Acceptance sends the segment to quadrant 3 (lower left) and brings the next entry from the highlighted fraction listing into the quadrant 2 plot. Spectral segments thus accumulate as an overlay in quadrant 3 until the highlighted XIC fractions listing is exhausted for a given peptide/timepoint. Overlaid spectral segments can then be mass-normalized to the average for all (to correct for very small differences in internally calibrated mass), then spectral peaks in the cluster are detected via a statistical algorithm, followed by weighted mean (centroid mass) calculation from the areas of all detected isotope peaks for each individual segment. The overlaid segments can then be averaged in quadrant 3 (with baseline offset correction), followed by spectral peak detection and weighted mean calculation for the averaged peak cluster. A button then sends the weighted means to the developing plot of centroid mass vs. time in quadrant 4 (plotted between F1H and F2H asymptotes), and this operation brings the XIC/first spectral segment for the next timepoint into quadrants 1 and 2 respectively. Upon completion of all timepoints, the rates plot data are output and spectral processing for a subsequent peptide can be initiated. All rates data from an experiment (averaged segment and all individual segments contributing to the average) are output to an experiment-specific "rates archive" workbook.

Additional features of "TOF2H-Editor" include: Ability to choose from multiple searches in the Processor output file and to save/restore partially edited experiments at the point at which editing was interrupted, as well as: **Quadrant 1**: (**1**) Dual-pass XIC construction with user-settable tight error tolerance for internally calibrated spots, and looser error tolerance for spots whose internal calibration had failed (Additional file 1: Footnote 2). (**2**) Overlayable XICs for isotope progeny (above). (**3**) "Absolute" and/or "relative" XICs, the latter calculated relative to peak heights for internal MALDI mass calibrants (XICs for the XIC for the calibrants themselves can also be viewed). **Quadrant 2**: (**1**) Semi- and fully-automated decision-making, namely approval/rejection of successive, above-noise segments across a displayed XIC, via a statistical algorithm. (**2**) Option to "snap" spectra to the internally calibrated peaklist-derived mz value (in case of calibration loss during download). (**3**) DefCal alert. **Quadrant 3**: Rejection of individual segments; grouping of segments for individual centroiding. **Quadrant 4**: (**1**) Calculation and display of BE rates from BE timepoints for each peptide in the experiment via linear regression and correction of the main rates plot accordingly. (**2**) Calculation and overlay of various theoretical horizontal asymptotes, on the fly, on the developing quadrant4 exchange rate charts. (**3**) Overlay of averaged spectral segments, from timepoint-to-timepoint, in a third quadrant 4 tab, with save in ASCII text format that is compatible with Weis & Engen's HX-Express macro. **Quadrants 1–3**: Vertical guidelines over charts marking F1H theoretical monoisotopic mass (quadrant 2, pink), current XIC fraction or F1H experimental monoisotopic mass (red), or isotope progeny mass if such a peak is being edited (quadrant 2, green). **Quadrants 2 and 3**: Quick adjustment of spectral endpoints (using sliders or entering values); restoration to initial values, single-click zoom-out and restore to check for interfering clusters. **All quadrants**: Single-click chart zoom functions with full functionality in the zoomed state.

### TOF2H-Rates Browser

**Figure 6:**
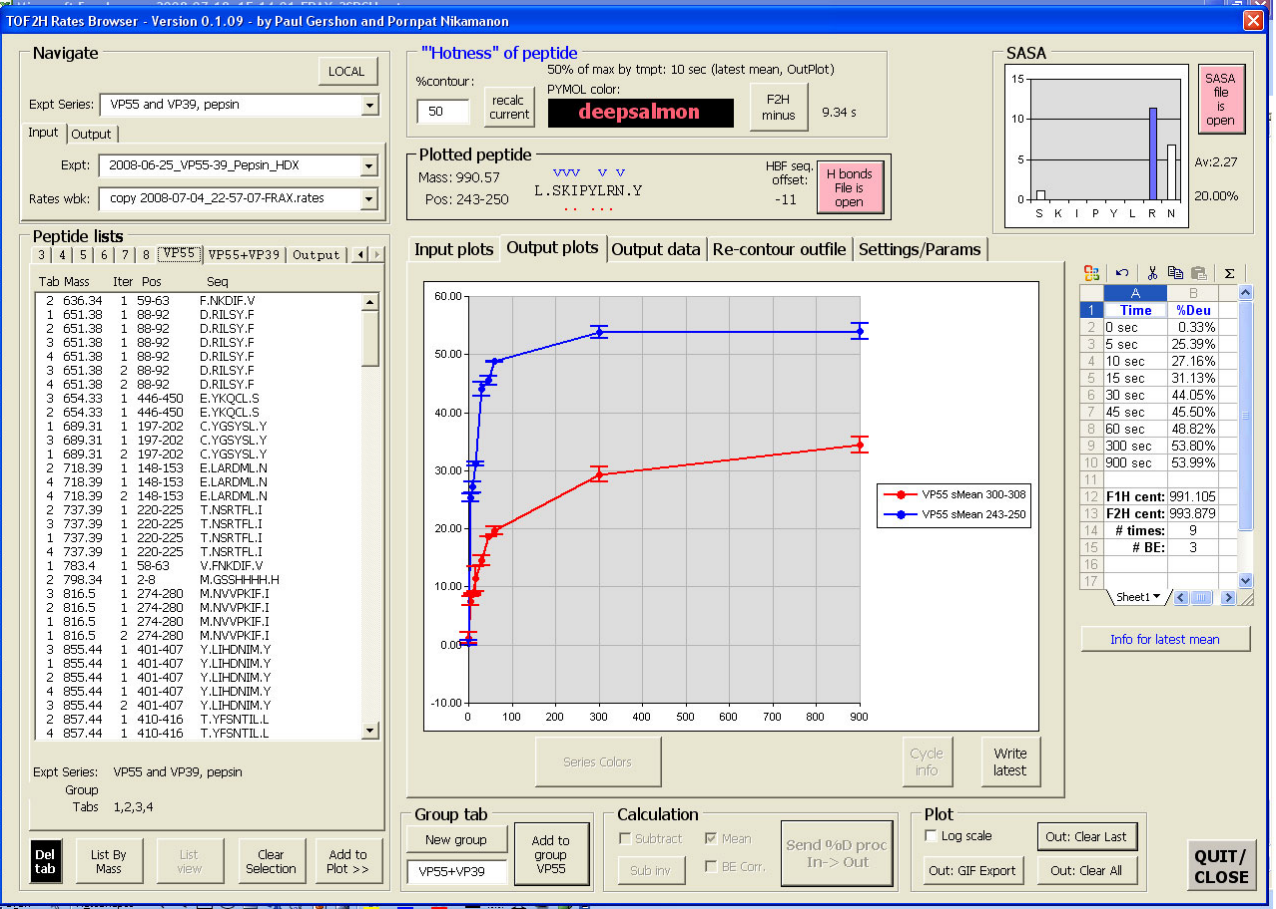
**Screen capture of TOF2H-Rates Browser.***"Navigation" frame*: Input "Rates" files data. *"Peptide lists" *frame, *"Group tab" *frame: Peptide picking (multi-tabbed by input file or file-group). *"Hotness" frame*: Suggested color for 3D visualization. "*Displayed peptide" frame*: Peptide info (red dots = theoretically exchangeable protons based on primary structure considerations only; blue "v" = hydrogen bonded amide protons predicted from crystal structure). *Central charts and "Calculation" frame*: Rate plot display, manipulation, and send to output chart. *"SASA" frame*: Main chain amide proton predicted solvent exposure as calculated by SASA [12].

TOF2H-Rates Browser (Fig. 6) is a processing and presentational tool for combining comparing rates plots between experiments. It takes, as input, EDITOR "rates archive" data (though it should be equally compatible with rates data generated by other software packages), from which the centroid masses of individual fractions, or scans, across an XIC peak are used to generate statistical populations. Currently, Rates Browser can: (**1**) Simultaneously list peptides from any number of input files, pool listings for replicate experiments under "group tabs", sort listed peptides by mass or sequence position with ability to switch between list-view and sequence-contig-view. (**2**) Overlay rates plots for any number of listed input peptides with or without BE correction and theoretical horizontal asymptotes, with separate BE rate plot overlays. (**3**) Convert Y-axis between various scales including centroid mass, % of maximum theoretical deuteration, % of maximum experimental deuteration, # of deuterons, convert X-axis between either log or linear, with selectable plot legend type. (**4**) Optionally interpolate missingtimepoint values. (**5**) Generate output chart as mean of all currently displayed input plots (either mean of individual XIC fraction centroid masses or mean of summed XIC peak centroids) with standard deviation error bars based on the populations for individual fractions, tabulating the resulting data and saving to an output file that includes an operations log. Any number of such plots can be overlaid in the output chart. (**6**) Re-import analyses from an output file for subtraction of pairs of mean rates plots. (**7**) Show the sequence of a currently plotted peptide marked-up with theoretically exchangeable amide protons and amide protons that are hydrogen bonded within an X-ray crystal structure. (**8**) Show SASA [12] plot, based on an X-ray crystal structure, of predicted solvent accessibility for each residue in the currently displayed peptide and calculate a mean SASA value across all exchangeable residues of the peptide. (**9**) Calculate the "transition" timepoint and interpolated "transition" time corresponding to the %deuteration level that attains, or surpasses, a user-defined "contour" value, and pick a corresponding color for 3D visualization, based on the transition timepoint, from a user-defined color vs. timepoint series. (**10**) Simultaneously "re-contour" all analyses in an output file to find the %deuteration contour that yields an optimum spread of color values for 3D visualization. (**11**) Plot mean SASA value vs. "transition" time for all analyses in an output file, and "re-contour" this plot. (**12**) Export GIF images of all charts with plots colored by series number or by 3D color value, or flattened to grey shades with selectable line/marker styles.

## Conclusion

The functions described form a core package of interconnected programs ("Solvent "Explorer/TOF2H") for the semi-automated processing and analysis of HDX data generated via an LC-MALDI workflow, from raw spectra and instrument-generated peaklists through BE-corrected, combined deuterium uptake rate plots. Additional modules and capabilities (eg. for LC solvents analysis) have not been described. In the standard TPF2H workflow, an experiment series (specific to a target protein or group thereof + peptidase) initiates with iterative LC-MS/MS analyses of peptidase digests of the protein/under fully protic conditions until growth of the resulting non-redundant library of MS/MS-confirmed peptide masses becomes asymptotic. TOF2H then chaperones instrument data from HDX experiments through a series of steps initiating with the generation of an experiment template, assembly of the contents of ~2700 or more individual instrument-derived spectral mass/height peaklists into a single data array containing 168,000 or more masses, then filtering of the array and alignment of equivalent masses, peptide library searching, and systematic processing of spectral segments for each "hit" peptide in turn.

TOF2H was designed with the nanoflow rates of LC-MALDI in mind. We are aware of just four reports in which HDX has been done at nanoflow rates [13] (all of which were nano-ESI as opposed to nanoLC-MALDI). If nanoflow approaches grow in popularity, specific issues may come into play such as variablility in chromatographic elution time ("dead time") due to the amplification of the effects of run-to-run differences in dead volume at low flow rates. This could provide a challenge for the "fixed box" spectral editing approach [6] in which HDX experimental spectra are edited on the basis of library peptide elution times. The *ab initio *approach employed by TOF2H has proven, in our nanoLC-MALDI experiments, resistant to dead-time effects, especially when combined with additional peptide validation and filtering on the basis of LC elution profile (data not shown). TOF2H is being upgraded for general instrument (mzML) compatibility and, in this regard, the LC-MALDI approach may be adaptable to the simpler MALDI-TOF instrument in place of the MALDI-TOF/TOF instrumentation reported here. Since TOF2H accepts database search results in standard format, MS/MS-confirmed peptide library construction could be performed on any ESI instrument in standard configuration followed by HDX experiments via MALDI-TOF. Such a "divorced" analysis may avoid the need for HDX-specific modifications to ESI mass spectrometers (such as the substitution of a delicate nanospray source for an ESI source that may be cooled and/or required only for HDX work) [14]. For this dual-instrument strategy to be effective, however, a MALDI-TOF with reasonably fast batch-acquisition rates would be required.

A significant amount of functionality is incorporated into the TOF2H toolset, whose performance has proven to be quite precise and robust. TOF2H matured with some elements in common with "The Deuterator" (see introduction), as may be inevitable due to the systemic nature of segments of the workflow. However, many features seem to be unique: TOF2H data processing workflow incorporates real-time verification, via interactive (semi-automated) spectral editing, as opposed to the more fully-automated data processing approach employed by "The Deuterator" which then requires manual validation as a follow-up. TOF2H takes an *ab initio *approach to isotope cluster finding in spectra, and XIC peak finding in chromatograms, as opposed to boxing predicted positions in the LC-MS spectral stacks (above). The *ab initio *approach involves scanning of the spectrometer software-generated, partially declustered peaklists for target peptides of interest prior to any spectral editing operations, then picking extant chromatographic peaks based on an examination of each XIC from beginning to end. Within the active fractions of an XIC, TOF2H verifies each spectral segment for the presence of a recognizable, well-segregated cluster prior to sending the spectral segment for summing. Thus, every segment that is summed and centroided has already been automatically or visually pre-validated in multiple steps. TOF2H is distinct in other ways too: It works through an entire HDX experiment from an experiment template file, it can provide an approximation of the extent of deuterium uptake over the experiment from peaklist analysis alone (prior to any spectral analysis), and during searches of MS/MS confirmed peptide lists it has the ability to find metal adducts and to reject mass matches that could be spurious matches to non-target proteins present in an experimental mixture. TOF2H has the capacity to display and process deuterium uptake plots, and it can calculate and implement BE corrections on the fly in a manner that normalizes for the potential influence of LC retention time on BE. Finally, the TOF2H package is portable, available for use on client PCs, in other labs, for desktop use, and is available from the authors at no charge.

In the general sense, HDX data processing strategies are likely to develop in an instrumentation-dependent manner, as a function of: (**1**) Instrument mass accuracy/resolution of spectral acquisition; (**2**) whether or not instrument software parses spectra into peaklists and whether these peaklists are partially or fully declustered; (**3**) whether sample input to the spectrometer is in the form of fractions or a continuous sample stream; (**4**) whether mass calibration is conferred experiment-wide or on a spectrum-by-spectrum basis; (**5**) how spectral resolution may impinge on the method used for centroid mass calculation or theoretical fitting [6]; (**6**) whether library hits and spectra are few enough to permit assessment/validation of each spectral segment in real time (the TOF2H approach) and whether validation is automated or done on a sampling basis only; (**7**) MALDI target plate layout; (**8**) chromatographic resolution; (**9**) Amount of BE that must be corrected, etc. We believe that within this landscape, TOF2H carves a distinct niche providing, to our knowledge, a number of capabilities that are not otherwise available. TOF2H has the potential, nonetheless, to be useful in a cross-platform manner without fundamental re-design.

TOF2H was implemented as an integrated series of macros in MS Excel/VBA (MS Excel 2003 or 2007 with Office Web Components) though it is now undergoing conversion to other languages. It runs in MS Windows XP or Vista environments, and can run either locally or remotely via a LAN. Data inputs comprise instrument-derived peaklist and raw spectrum files (ascii text), instrument database XML export files, database (eg. Mascot) search results exported as csv, and a user-filled LC Parameters spreadsheet. Intermediate and final data output are in the form of MS Excel spreadsheets. GIF files of processed deuterium uptake rates plots and other charts can be exported.

## Methods

### Initial MS/MS experiments

HDX analysis of a specific protein in conjunction with a specific protease, was initiated with the development of a preliminary accurate-mass reference "MS/MS-confirmed peaklist" (refMPL). Experimental details will be described in more detail elsewhere (Koter *et al*, in preparation). Briefly, the target protein was digested for various times with a non-pecific acid-protease (typically pepsin), then samples were mixed back and subjected to automated nanoLC-MALDI analysis, spotting robotically into 288 spots with online matrix dosage (including internal calibrant). The resulting MALDI target plate was then subjected to MS/MS batch analysis using the AB-Sciex 4700 MALD-TOF/TOF, in which instrument manufacturer's software determines XIC peak fractions (spots) across the plate for MS ions above a specified abundance threshold, then generates MS/MS spectra for all of these ions from their XIC peak fractions. The resulting MS/MS fragmentation data were sent to the Mascot search engine (; [8]) for fragment mass-matching to proteins within a specified database that had been digested to peptides by Mascot *in silico*, at all theoretical combinations of the protein's interpeptide bonds.

### HDX wet experiment

Details of HDX wet experiments will be described in more detail elsewhere (Koter *et al*, in preparation). Briefly, each such experiment is performed by diluting protein solution into deuteric buffer followed by incubation in this buffer for a preset time period (timepoint), then quenching with protic acid at close to 0°C. Controls included "F1H" (fully protic: Protic quench first, followed by protic buffer dilution), "0 sec" (protic quench first, followed by deuteric buffer dilution) and "F2H" (fully deuteric: Deuteric dilution followed by deuteric quench). Quenched samples were immediately loaded to a manually controlled, high resolution nanocapillary C18 LC column, then immediately eluted with a rapidly-developing (3 min) solvent gradient in 0.1% TFA, dosing the column output on line with MALDI matrix and internal calibrants, and manually depositing fractions directly onto the 192 etched spots of a chilled stainless steel MALDI target plate. The sample plate was then immediately subjected to < 50 mTorr of vacuum and immediately docked in the 4700.

### HDX timpoint data acquisition and recovery

Docking was immediately followed by a "batch" MS data acquisition session (Dock plate -> (acquire/store spectra)^n ^-> undock plate). MS spectra were acquired via 1000 laser shots and saved using "**4000 Series Explorer**" software in batch mode, employing an instrument-derived spectrum processing method that lists monoisotope peaks with S:N greater than a preset value and effects instrument calibration via the internal calibrants. After repetition of this process with different times of HD exchange, the data from each timepoint became associated with a unique TSQUARED-derived JRID. F1H plates were typically subjected, also, to MS/MS batch analysis (RESULTS). After data acquisition for all timepoint samples of an HDX experiment, all MS output peaklists and raw spectra from each relevant spotset were recovered from TSQUARED into stand-alone ASCII text files (one folder per spotset, Additional file 1), using the tool "TOF-TOF Data Extractor" (T2DE, ). In our hands, a 192-spot plate-full of MS spectra could be downloaded in approximately 5 – 8 min. The XML database export function of "4000 Series Explorer" software allowed manual dumping of all summary information for spotsets to XML files which were utilized by TOF2H for ETF generation and other functions.

## Abbreviations

amu: Atomic mass units; BE: Back exchange; Da: Dalton; DB: Database; ET: Experiment Template; .etf/ETF: Experiment template file; ET: Experiment template; F1H: Fully protic; F2H: Fully deuteric; GUI: Graphical User Interface; HDX: Hydrogen/deuterium exchange; ID: Identification; JRID: JOB_RUN_ID; LAN: Local Area Network; LC: Liquid Chromatography; LC-MALDI-TOF/TOF: Mass Spectrometry by LC MALDI with Time of Flight (dual-stage) analysis; MALDI: Matrix-Assisted Laser Desorption; MPL: MS/MS-confirmed peaklist; MS: Mass spectrometry; MS/MS: MS followed by selection/isolation of individual ions and MS of the fragments; m/z: Mass-to-charge ratio; NMR: Nuclear magnetic resonance; RefMPL: Reference MPL (containing one copy each of all MS/MS-confirmed peptide for the target protein, over a series of sheets); T2DE: T2 Data Extractor ; TFA: Trifluoroacetic acid; TOF: Time of flight; XIC: Extracted Ion Chromatogram.

## Authors' contributions

Initial discussions between PG and MK conceptualized the project and emphasized the key requirements for the processing of LC-MALDI data. PG then initiated the project, with conceptualization and design of all software modules. Of the five core modules, PG wrote "ETF-Generator". "MPL tools", "Assembler", approximately half of "Processor" and approximately two-thirds of "Rates Browser". MK discovered EP and NP as programmers. EP wrote the first generation of processor, encoding the key isotope analysis algorithms, with extensive external feedback and guidance from PG. MK provided test data, and extensive feedback during the debugging of early generations of Processor and Editor, including manual checking of deisotoping functions. PN encoded then developed Editor in its entirety, with extensive feedback and suggestions from PG and a few from MK. PN also wrote approximately one-third of "Rates Browser". PG wrote the manuscript in its entirety along with the conception and generation of all figures. PG provided overall support and supervision. Of the ~26,500 lines of code in the currently released version of TOF2H, ~9.5%, 33.5% and 57% were written by EP, PN and PG respectively. All authors read and approved the final version

## Supplementary Material

Additional file 1 **Supplementary information.** Click here for file
