# Supplementary material for: "TOF2H": A precision toolbox for rapid, high density/high coverage hydrogen-deuterium exchange mass spectrometry via an LC-MALDI approach, covering the data pipeline from spectral acquisition to HDX rate analysis
Source: BMC Bioinformatics. 2008 Sep 20;9:387. doi: 10.1186/1471-2105-9-387 (PMC2561049; doi:10.1186/1471-2105-9-387)
Supplement: Additional file 1 — Supplementary information. [file 1471-2105-9-387-S1.pdf]

Supplementary information

Supplementary Table 1: Data files used and generated by TOF2H

Filename suffix	Function	Format	Output by	Modified by
.pkl	Peaklist data files from individual spectra	ASCII Text	T2DE	-
.txt	Raw spectrum ASCII	ASCII Text (?)	T2DE	-
.etf	Experiment template file	MS Excel	TOF2H-ETF Generator	TOF2H-ETF Generator
.csv	Mascot data export	MS Excel	Mascot	-
.mpl	MSMS-confirmed peaklist file	MS Excel	TOF2H-MPL Curation Tools	TOF2H-MPL Curation Tools
.refMPL	Reference MPL	MS Excel	TOF2H-MPL Curation Tools	TOF2H-MPL Curation Tools
.ass	Consolidated peaklist data	MS Excel	TOF2H-Assembler	TOF2H-Assembler
.xls	Consolidated peaklist data prefiltering, padding, matching and post-processing	MS Excel	TOF2H-Processor	TOF2H-Processor
.edi	Spectrum editing, working file	MS Excel	TOF2H-Editor	TOF2H-Editor
.pep/.xls	Spectrum splice export	MS Excel	TOF2H-Editor	-
.rate	Archive of deuterium uptake rates	MS Excel	TOF2H-Editor	-

Supplementary Table 2: Mass tolerances used by TOF2H. “inter-spot” and “inter-plate” mass tolerances represent fractions from the same or distinct LC gradients (timepoints), respectively. The observed error when comparing internally calibrated observed masses between plates is a combination of internal and external calibration mass errors in which the external components cancel to zero. Therefore, with internal calibration, the two tolerances are theoretically identical. Since all masses in the experiment are internally calibrated, the plate of origin should therefore have no

theoretical impact on mass accuracy. In practice, however, the latter was typically set ~20% wider than the former to accommodate any inter-plate differences in internal calibration should they occur.

From Table	To Table	Used to:	By:	Same spot?	Same plate?	Same acq session?	Same expt?	Used m
15	20	Eliminate neutron ladder members	Finding rungs of neutron ladder	✔	✔	✔	✔	Neutron- mass tol
25	35	Find calibrant mass; find the spot with max peak height within XIC cluster.	Finding match to calibrant mass; finding XIC cluster borders (same mz, same timepoint, various %CH3CN)	✖	✔	✔	✔	Inter-spo toleranc
35	45	Group same mz peptide across different timepoints (to same row of table 3)	Finding timepoint cluster borders (same mz, different timepoints)	✖	✖	✖	✔	Inter-pla toleranc
50	55 (primary hit)	Find experimental peptides	Finding experimental hits to MPL	✖	✖	✖	✖	Inter-spo toleranc
50	55 (progeny hit)	Find neutron shifts with duration of HDX	Finding matches between experimental F1H mz and experimental neutron progeny from various HDX timepoints	✖	✖	✖	✔	Inter-pla toleranc

Supplementary Footnote 1 Each MPL sheet contains peptide sequences, start/end positions, experimental MH$^+$, experimental M, matching theoretical M, Mascot's scores) supplemented with additional data by TOF2H (namely calculated theoretical MH$^+$, theoretical max # of possible amide HD exchanges (peptide length minus 2 [16] minus the number of internal prolines), estimated preliminary ^{13}C isotope cluster width (peptide mass multiplied by an empirical value of ≈0.0044), estimated "Total Shiftable Bandwidth" (sum of the previous two values), MALDI spot identifiers, timestamp and file tracking information.

Supplementary Footnote 2 Unprocessed or filtered peaklist data are queried by the target peptide mass in two passes: The first for internally calibrated (tight error tolerance) matches to masses in spots passing internal calibration, the second pass supplements the first with masses from internal calibation fail spots (deduced from the ET) but within a wider mass tolerance. In order to permit XIC construction in the context of deuteric shifting, the above process is repeated with any neutron progeny masses listed for the timepoint/mass in the search results.

Supplementary Figure 1: Current TOF2H directory structure. Within the “Data” directory, experiment series-level directories (eg. “VP55 and VP39, pepsin”) contain groups of experiments (eg. “2008-03-04-VP55-capillary”) that share target proteins and protease (and therefore contain common refMPL and associated MPL and csv files). Folders ending “_HDX” contain Instrument dumps of output peaklists and raw spectra. Partially edited experiments are saved in the “Editor files” directory for later resumption. “Edited splices” contains compiled spectral splices in a format compatible with Weis & Engen’s “HX-Express”.

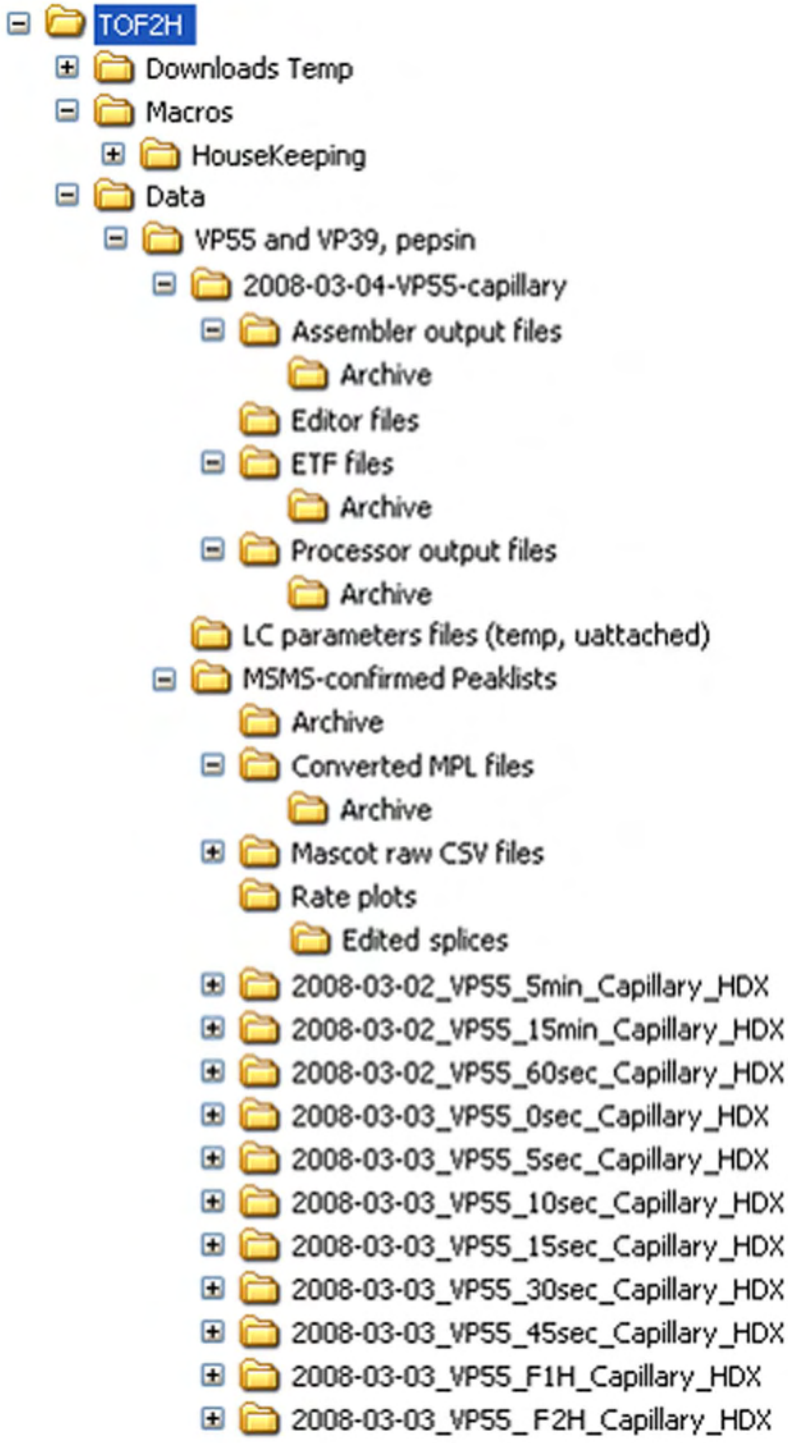
TOF2H
Downloads Temp
Macros
HouseKeeping
Data
VP55 and VP39, pepsin
2008-03-04-VP55-capillary
Assembler output files
Archive
Editor files
ETF files
Archive
Processor output files
Archive
LC parameters files (temp, uattached)
MSMS-confirmed Peaklists
Archive
Converted MPL files
Archive
Mascot raw CSV files
Rate plots
Edited splices
2008-03-02_VP55_5min_Capillary_HDX
2008-03-02_VP55_15min_Capillary_HDX
2008-03-02_VP55_60sec_Capillary_HDX
2008-03-03_VP55_0sec_Capillary_HDX
2008-03-03_VP55_5sec_Capillary_HDX
2008-03-03_VP55_10sec_Capillary_HDX
2008-03-03_VP55_15sec_Capillary_HDX
2008-03-03_VP55_30sec_Capillary_HDX
2008-03-03_VP55_45sec_Capillary_HDX
2008-03-03_VP55_F1H_Capillary_HDX
2008-03-03_VP55_ F2H_Capillary_HDX
